# Implicit Health Beliefs and Suicidal Ideation in Chinese College Students: The Serial Mediating Roles of Lifestyle Behaviors and Anxiety Symptoms

**DOI:** 10.3390/bs16091619

**Published:** 2026-09-10

**Authors:** Xinxin Lai, Jinghong Huang, Honglin Yan, Zhenkun Shi, Yuxin Zhang, Yongfu Yang, Xiaojun Liu

**Affiliations:** 1 Department of Social Medicine and Health Management, School of Health Management, Fujian Medical University, No. 1 Xuefu North Road, University New District, Fuzhou 350122, China; xinxin_lai@fjmu.edu.cn (X.L.); jinghong.hua@student.mahidol.ac.th (J.H.); yanholy@fjmu.edu.cn (H.Y.); shizk_2003@fjmu.edu.cn (Z.S.); 19103229965@fjmu.edu.cn (Y.Z.); yongfuyang@fjmu.edu.cn (Y.Y.); 2 Department of Society and Health, Faculty of Social Sciences and Humanities, Mahidol University, Nakhon Pathom 73170, Thailand; 3 The Key Laboratory of Environment and Health of Fujian Province University, Fujian Medical University, Fuzhou 350122, China; 4 NHC Key Laboratory of Etiological Epidemiology of Chronic Diseases with High Incidence in Fujian-Taiwan Area (Co-Construction), Fujian Medical University, Fuzhou 350122, China

**Keywords:** implicit health beliefs, suicidal ideation, lifestyle behaviors, anxiety symptoms, serial mediating roles

## Abstract

The growing prevalence of mental health problems and suicidality among university students highlights the need to identify potentially modifiable psychological pathways that can inform preventive interventions. This study investigated the association between implicit health beliefs and suicidal ideation among Chinese college students and examined whether lifestyle behaviors and anxiety symptoms jointly explained this relationship. A cross-sectional survey was conducted among 1964 college students using the Lay Theories of Health Scale, 24-H Movement Behaviors Questionnaire, 7-item Generalized Anxiety Disorder, and Beck Suicide Ideation Scale. Serial mediation analyses were performed to test the indirect effects of lifestyle behaviors and anxiety symptoms. Implicit health beliefs were directly associated with lower suicidal ideation (direct effect: −0.0562, 95% CI: −0.0767 to −0.0357). In addition, lifestyle behaviors (indirect effect: −0.0049, 95% CI: −0.0086 to −0.0015), anxiety symptoms (indirect effect: −0.0644, 95% CI: −0.0770 to −0.0523), and their serial mediation pathway (indirect effect: −0.0018, 95% CI: −0.0035 to −0.0002) significantly mediated this association, accounting for 55.82% of the total effect. These findings suggest that implicit health beliefs may be associated with suicidal ideation, with lifestyle behaviors and anxiety symptoms statistically accounting for part of this association. Targeting these modifiable potential pathways may help inform the development of theory-driven psychological interventions to reduce suicidal ideation and promote mental health among higher education students.

## 1. Introduction

Suicide is a critical public health concern, representing not only a profound personal tragedy but also a significant social and economic burden (Cerel et al., 2019; Gavan et al., 2026). Globally, suicide is the second leading cause of death among individuals aged 15–29 years (Xuan et al., 2023). A large meta-analysis involving more than 634,000 students estimated that the pooled lifetime prevalence of suicidal ideation was 22.3% and that of suicide attempts was 3.2% (Mortier et al., 2018). Suicidal ideation encompasses thoughts, plans, and intentions (Large et al., 2021) related to suicide and serves as a key predictor of subsequent suicidal behavior, making it a critical focus for prevention strategies (Mahumud et al., 2022).

Previous studies have identified a range of factors associated with suicidal ideation, including demographic characteristics such as gender and age (Carretta et al., 2023; Huang et al., 2022), health-related behavioral factors such as alcohol consumption (Seo et al., 2021), and psychological and psychosocial factors such as psychological resilience, emotional coping and affective disorders (Gao et al., 2025; Huang et al., 2022; Okechukwu et al., 2022; Tabares & Restrepo, 2025). In addition to these factors, attitudes toward suicide may also be associated with suicidal ideation. A permissive attitude toward suicide refers to the permissive and acceptable attitudes toward suicide, considering it a right or option in certain circumstances, such as incurable diseases (Hong et al., 2024). Previous studies have shown that the more permissive attitude toward suicide, the more severe the suicidal ideation (Lee et al., 2021). However, most existing research has focused on explicitly reported psychological factors or attitudinal factors directly related to suicide; these factors may be influenced by stigma, concealment and cultural barriers to disclosure—particularly in the Chinese context, where discussing personal or family psychological difficulties is often discouraged (Wang et al., 2022). These limitations highlight the importance of examining latent health beliefs that may be associated with suicidal ideation.

In recent years, there has been growing interest in implicit theories of health—beliefs about whether health is fixed or malleable. These beliefs are typically understood in terms of two dimensions: firstly, the entity theory of health, which holds that health is relatively stable and not easily influenced by individual factors; and secondly, the incremental theory of health, which holds that health is malleable and that individuals can improve their health through effort and behavioral change (Schreiber et al., 2020). Importantly, these beliefs should be distinguished from the Health Belief Model, which primarily explains specific health-related behaviors through constructs such as perceived susceptibility, perceived severity, perceived benefits, and perceived barriers (Janz & Becker, 1984). By contrast, implicit health beliefs represent broader assumptions held by the public regarding whether individual health is relatively fixed or malleable and controllable; they focus more on the perceived malleability of health itself, rather than on cognitions relating to specific diseases or health behaviors. Empirical research supports this theoretical pathway: stronger adherence to the incremental theory of health is associated with more positive health attitudes and health behaviors. A randomized smartphone-based intervention study further found that enhanced progressive health beliefs were associated with greater engagement in health-promoting behaviors in everyday life (Schreiber & Dohle, 2023). Related research on implicit theories also suggests that beliefs oriented toward health plasticity may be associated with more positive psychological and perceived physical health outcomes (Schreiber & Dohle, 2023; Valdez, 2024). Taken together, these findings suggest that implicit health beliefs may be associated with health-related behavioral patterns as well as psychological and health outcomes.

Lifestyle behaviors, including physical activity, sedentary behavior, and sleep, are recognized as the three pillars of a healthy 24 h movement framework (Shiran & Nigg, 2024). Evidence suggests that adherence to these lifestyle behaviors is associated with reduced risk of suicidal ideation (García-Hermoso et al., 2022) and high health literacy (Ghorbani-Dehbalaei et al., 2021). And compared to a single unhealthy behavior, the occurrence of multiple unhealthy lifestyle behaviors shows stronger associations with suicidal ideation and suicidal behavior (Lai et al., 2024). Furthermore, anxiety has been well-established as a risk factor for suicidal ideation, making it reasonable to advocate for the inclusion of anxiety measures at the symptomatic level when assessing suicide risk (Grant et al., 2023). Moreover, research indicates that factors predicting anxiety include lower beliefs in healthy lifestyles and fewer healthy lifestyle behaviors (Hoying et al., 2020). Consequently, these findings suggest that lifestyle behaviors and anxiety symptoms may act as mediating factors in the relationship between implicit health beliefs and suicidal ideation.

**Figure 1:**
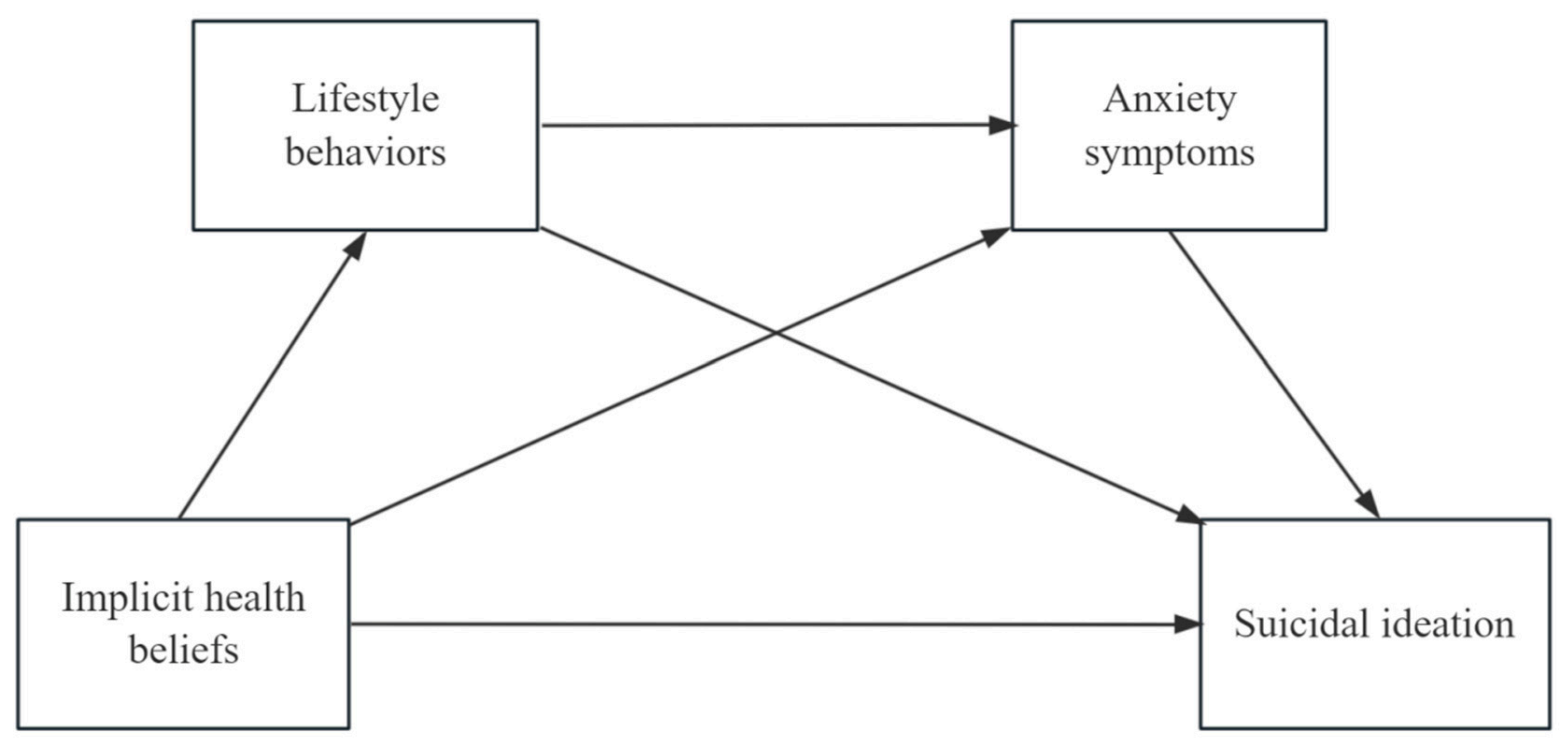
Hypothesized serial mediation hypothetical model.

Accordingly, drawing on social cognitive theory, the two-system model, and the stress-susceptibility framework, this study aims to test a mediational model that provides theoretical support for the relationship between implicit health beliefs and suicidal ideation among college students. Specifically, we examined three indirect pathways: (1) the mediating role of lifestyle behaviors, (2) the mediating role of anxiety symptoms, and (3) a sequential mediating pathway from implicit health beliefs to suicidal ideation via lifestyle behaviors and anxiety symptoms (Figure 1).

## 2. Methods

### 2.1. Study Design and Population

The authors assert that all procedures contributing to this work comply with the ethical standards of the relevant national and institutional committees on human experimentation and with the Helsinki Declaration of 1975, as revised in 2013. All procedures involving human subjects/patients were approved by the Biomedical Research Ethics Review Committee of Fujian Medical University (Approval No. 264 [2024]). Written informed consent was obtained from all subjects.

We employed a cross-sectional survey design. Participants were recruited through stratified sampling by undergraduate year and major, with recruitment targets for each “year × major” stratum determined proportionally to its share of the total undergraduate population. We endeavored to maintain these proportions during data collection. However, due to voluntary participation and convenience sampling within each stratum, the final valid sample did not fully reflect the enrollment distribution. The actual number of registered students, the target proportions by major and grade level, the final valid sample size, and the actual proportions by major and grade level are presented in Table S1. Based on a total undergraduate student population of 10,438, and using standard survey sampling methods (assuming a 95 per cent confidence level and a 5 per cent margin of error), the minimum required sample size, after adjustment for a finite population, was calculated to be approximately 371 valid respondents; allowing for a 20 per cent non-response or invalid response rate, the sample size was approximately 464. As our aim was to maximize coverage across all grades and majors by recruiting as many eligible students as possible, we distributed a total of 1999 questionnaires, of which 1964 valid responses were retained (951 online responses and 1013 offline responses), yielding a valid response rate of 98.25%. All items in the online questionnaire are marked as required. However, since it is not technically possible to enforce responses in paper-based surveys, some paper questionnaires contain unanswered items. Missing values were addressed using multiple imputation, and five imputed datasets were generated for subsequent analyses.

Data collection took place between October and November 2024, utilizing both online (Questionnaire Star) and offline (paper-based) survey methods. Paper questionnaires were distributed during break times and collected once completed; online questionnaires were accessed via a QR code and submitted independently by respondents. Prior to participating in the survey, students received a standardized information sheet regarding the purpose of the study, voluntary participation, confidentiality and informed consent, and were informed that they should not participate again if they had previously completed the survey. Participants were undergraduate students from various faculties and disciplines at a comprehensive medical university in Fujian Province, China.

The scales were administered in a fixed order. Participants first completed the Lay Theories of Health Scale, followed by the 24-H Movement Behaviors Questionnaire (24HMBQ) with the Beck Suicide Ideation Scale interspersed within it, and finally 7-item Generalized Anxiety Disorder Scale (GAD-7). Because the content of the Beck Suicide Ideation Scale is relatively sensitive, it is embedded within other scales rather than placed at the beginning of the questionnaire, in order to help minimize potential priming effects.

Inclusion Criteria: Participants must be full-time undergraduate students at the university and voluntarily sign an informed consent form prior to completing the questionnaire.

Exclusion criteria: A questionnaire is excluded if any of the following conditions apply: (1) missing data exceeding 10% of items; (2) evident patterned responses (e.g., selecting the same option for all items); (3) incorrect responses to quality control questions; (4) completion within an unusually short timeframe (less than 3 min).

### 2.2. Measures

Implicit Health Beliefs: Three items adapted from the Chinese version of the “Lay Health Theory Scale” were used to assess implicit health beliefs. This scale was originally developed by Bunda and Busseri to assess lay theories regarding health plasticity and fixity (Bunda & Busseri, 2019). The Chinese version used in this study is based on an adaptation developed by Da et al. (2022) for Chinese adults. We adopted two entity-belief items (“Health is a part of me that I cannot change” and “I can maintain my health in certain ways, but I cannot change my basic health status”) and one incremental-belief item (“Regardless of my current health status, I can take action to change it”). Items were scored using a 5-point Likert scale (1 = strongly disagree, 5 = strongly agree). Two of the entity belief items were reverse-coded, and the item scores were summed; a higher score indicates a stronger incremental belief in health plasticity (Tang et al., 2024). The scale assesses current or general beliefs regarding whether health is fixed or plastic, with no specific time frame for recall. The Cronbach’s α coefficient was 0.732. Exploratory factor analysis supported a unifactorial structure (KMO = 0.608; Bartlett’s test: $x^{2}$ = 1682.078, *p* < 0.001), with factor loadings ranging from 0.428 to 0.779.

Lifestyle Behaviors: We used the 24HMBQ to measure this variable (Zheng et al., 2023). This scale is used to assess physical activity, sedentary behavior, and sleep over the past week. A formative index was developed to assess adherence to three independent lifestyle recommendations based on established tools and guidelines (Lin et al., 2024; Luo et al., 2025; Ross et al., 2020). For each domain, participants were coded as 1 if they met the predefined recommendations and 0 otherwise. For physical activity, participants were considered compliant if they reported ≥150 min of moderate-to-vigorous physical activity per week and engaged in strength training ≥ 2 times per week. Compliance with sedentary behavior was defined as ≤8 h of sitting time per day and ≤3 h of recreational screen time per day. Sleep adherence was defined as 7–9 h of sleep per day. These three binary indicators were summed to yield a total lifestyle behavior adherence score ranging from 0 to 3 points. A higher score indicated greater adherence to more lifestyle recommendations.

Anxiety Symptoms: Measured using the GAD-7 with responses on a 4-point scale (from 0 = not at all to 3 = nearly every day) (Hua et al., 2025). Scores ranged from 0 to 21, with higher values indicating more severe anxiety (Wu et al., 2024). This scale assesses the frequency of anxiety symptoms over the past 2 weeks. In this study, the Cronbach’s α coefficient for the scale was 0.940.

Suicidal ideation: Assessed using three items (“Over the past week, how would you rate your desire to live?”, “Over the past week, how would you rate your desire to die?” and “Over the past week, what have you thought about when considering your reasons for living or dying”) from the Beck Suicide Ideation Scale (McCall et al., 2021). Each item was rated 0–2, with higher scores reflecting stronger suicidal ideation. This scale assesses suicidal ideation over the past week. In this study, the Cronbach’s α coefficient for the scale was 0.642. Exploratory factor analysis supported a unifactorial structure (KMO = 0.606; Bartlett’s test: $x^{2}$ = 859.483, *p* < 0.001), with factor loadings ranging from 0.454 to 0.702.

Covariates: Gender (male and female), grade (freshman, sophomore, junior, and senior), academic discipline (medicine, science or technology, and management or law), residence (natural village, township, county township, main city of prefecture), monthly living expenses (≤1000 Yuan, 1001~1500 Yuan, 1501~2000 Yuan, 2001~2500 Yuan, and >2500 Yuan), and average monthly household income (<2000 Yuan, 2000~4999 Yuan, 5000~9999 Yuan, and ≥10,000 Yuan).

### 2.3. Statistical Analyses

Missing data for the main variables were addressed using multiple imputation (4 missing values for implicit health beliefs, 280 for suicidal ideation, 259 for lifestyle behaviors, and 9 for anxiety symptoms), and five imputed datasets were generated for subsequent analyses. All subsequent analyses used the imputed data. Participants’ characteristics were described with a number (percentage). One-way analysis of variance (ANOVA) was used to test differences in suicidal ideation scores across different demographic groups. Correlation analyses used Spearman coefficients. The covariates included in the mediation analysis were gender, grade level, academic discipline, residence, monthly living expenses, and average monthly household income. For categorical variables, the reference categories were freshman, medicine, natural village, monthly living expenses ≤ 1000 Yuan, and average monthly household income < 2000 Yuan, respectively. Implicit health beliefs were set as the independent variable, lifestyle behaviors and anxiety symptoms as mediating variables, and suicidal ideation as the dependent variable; the covariates were included in the corresponding regression equations. Serial mediation models were estimated using PROCESS macro (Model 6) in SPSS 26.0, with 5000 bootstrap samples to compute 95% bias-corrected confidence intervals. Indirect effects were considered significant when CIs did not include zero.

All statistical analyses were conducted using SPSS 26.0. Two-tailed *p* < 0.05 was set as the threshold for statistical significance.

## 3. Results

### 3.1. Descriptive Analysis

This study included 1964 participants, ranging in age from 18 to 28 years. A higher proportion of the participants were females, freshmen, medical majors, and those residing in the main city of the prefecture. Table 1 showed more participants reported monthly living expenses in the range of 1501~2000 Yuan and average monthly household income in the range of 5000~9999 Yuan. Significant differences in suicidal ideation scores were observed across groups differing in gender, grade, academic discipline, residence, monthly living expenses, and average monthly household income (*p* < 0.05). Post hoc Tamhane’s T2 tests further identified significant pairwise differences in suicidal ideation across several sociodemographic characteristics. For grade level, significant differences were observed between freshmen and sophomores and between freshmen and juniors. For academic discipline, a significant difference was observed between medicine and science/technology students. For residence, significant differences were observed between natural village and township, natural village and county township, and natural village and main city of prefecture, as well as between township and county township and between township and main city of prefecture. For monthly living expenses, significant differences were observed between ≤1000 Yuan and each of the other four categories (1001~1500 Yuan, 1501~2000 Yuan, 2001~2500 Yuan, and >2500 Yuan), as well as between 1001~1500 Yuan and each of the remaining three higher-income categories (1501~2000 Yuan, 2001~2500 Yuan, and >2500 Yuan). For average monthly household income, significant differences were observed between <2000 Yuan and each of the three higher-income categories (2000~4999 Yuan, 5000~9999 Yuan, and ≥10,000 Yuan).

### 3.2. Correlation Analysis

Implicit health beliefs were positively associated with healthy lifestyle behaviors (ρ = 0.198, *p* < 0.01) and negatively with anxiety symptoms (ρ = −0.340, *p* < 0.01) and suicidal ideation (ρ = −0.266, *p* < 0.01). Lifestyle behaviors were negatively correlated with anxiety symptoms (ρ = −0.160, *p* < 0.01) and suicidal ideation (ρ = −0.166, *p* < 0.01). Anxiety symptoms were strongly positively correlated with suicidal ideation (ρ = 0.471, *p* < 0.01) (Table 2).

### 3.3. Mediation Analysis

**Figure 2:**
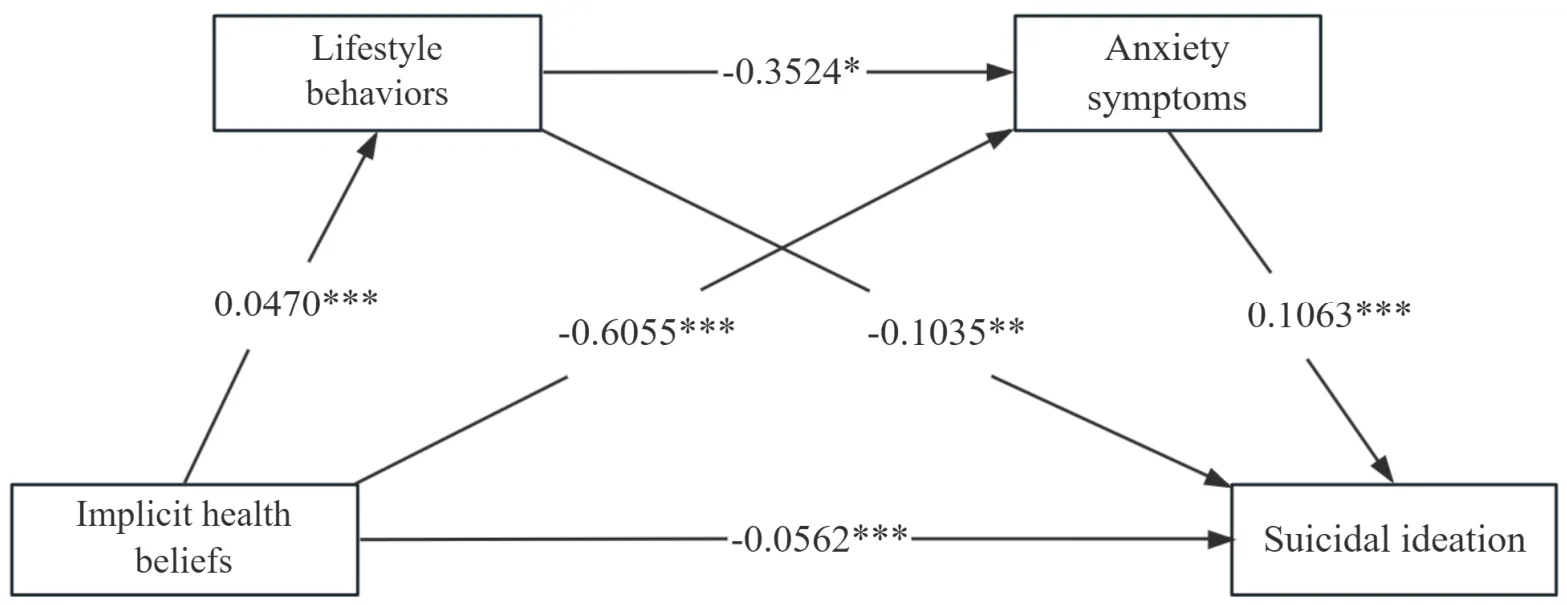
Estimated serial mediation model linking implicit health beliefs with suicidal ideation. * *p* < 0.05, ** *p* < 0.01, *** *p* < 0.001.

Regression analyses showed that implicit health beliefs were negatively associated with suicidal ideation (B = −0.0562, *p* < 0.001). The mediation analysis showed statistically significant indirect associations through three pathways: through lifestyle behaviors, through anxiety symptoms, and sequentially through lifestyle behaviors and anxiety symptoms. The indirect pathway through anxiety symptoms accounted for 50.63% of the total association, representing the largest indirect pathway, whereas the pathways through lifestyle behaviors and sequentially through lifestyle behaviors and anxiety symptoms accounted for 3.85% and 1.42%, respectively. Overall, the three indirect pathways accounted for 55.82% of the total association between implicit health beliefs and suicidal ideation (Table 3 and Table 4, Figure 2).

## 4. Discussion

This study found that implicit health beliefs were significantly associated with suicidal ideation among Chinese college students, with statistically significant direct and indirect associations involving lifestyle behaviors and anxiety symptoms. The findings underscore the intertwined roles of cognition, behavior, and their association with suicide prevention.

### 4.1. The Direct Effect of Implicit Health Beliefs on Suicidal Ideation

This study found that stronger incremental health beliefs are associated with lower levels of suicidal ideation. Implicit health beliefs reflect general views on whether health is relatively fixed or can be changed through personal effort, rather than specific attitudes toward particular health behaviors. Individuals who view health as highly malleable may be more likely to perceive health-related difficulties as manageable and maintain a stronger sense of personal agency, which helps them engage in adaptive self-regulation and coping when facing health challenges (Jia et al., 2024; Tang et al., 2024). Conversely, weaker incremental health beliefs may be associated with a perception of limited control over health outcomes, which may increase psychological vulnerability when facing adverse experiences. This finding is consistent with previous research indicating that more adaptive health-related beliefs and attitudes are associated with lower levels of suicidal ideation (Large et al., 2021). Such beliefs may influence how individuals assess health challenges, perceive their own ability to influence outcomes, and adopt adaptive coping strategies. However, these explanations are currently only theoretically plausible and have not yet been established as definitive mechanisms.

### 4.2. Mediating Effects of Implicit Health Beliefs Through Lifestyle Behaviors

First, implicit health beliefs can motivate health-related behaviors including exercise, healthy eating, and sleep (Thomas et al., 2019). This study categorizes lifestyle behaviors into three modules: physical activity, sedentary behavior, and sleep. The effects of physical activity may be explained by underlying physiological mechanisms. Studies have shown that physical activity improves mitochondrial function, promotes angiogenesis and synapse formation, and thereby helps alleviate negative emotional issues (Baskerville et al., 2023), which may play an important role in suppressing suicidal thoughts. Existing research has demonstrated that sedentary behavior increases the risk of suicidal ideation (Yu et al., 2024). Sleep deprivation is associated with negative mental health outcomes such as mood deficits (Short et al., 2020) and impulsivity (Porras-Segovia et al., 2019), potentially increasing suicidal ideation risk. Sleep disorders have also been identified as a predictor of suicidal ideation (Cox et al., 2023).

Secondly, from the perspective of information processing, Hofmann et al. proposed the two-system model, which posits that self-control success depends on the interaction between the impulse system and the control system (Hofmann et al., 2009). The emergence of suicidal thoughts is similarly associated with self-control capacity. Stronger incremental health beliefs enhance the functioning of the control system, promote healthy lifestyle behaviors, and thereby inhibit suicidal impulses.

### 4.3. Mediating Effects of Implicit Health Beliefs Through Anxiety Symptoms

The stress-susceptibility model suggests that suicide is the result of the interaction and joint action between stress factors, protective factors and individual qualities (Zhu et al., 2020). When an individual becomes susceptible and is in a state of stress, they may activate a state that makes them have suicidal ideation. Individuals holding lower incremental health belief characterized by immutability perceive health issues as “uncontrollable threats” rather than “solvable challenges”. Thus, lower incremental health belief serves as a vulnerability factor. When confronted with health-related stressors, susceptible individuals may be triggered into a state of stress, experiencing negative emotions such as frustration and anxiety symptoms. Research confirms a positive predictive relationship between mental health issues like anxiety symptoms and suicidal ideation (Cifrodelli et al., 2024). High levels of anxiety symptoms may exacerbate emotional exhaustion and impulsive behavior, ultimately contributing to the formation of suicidal ideation (Cheng et al., 2022). Furthermore, given that this mediating path made a relatively significant contribution in this study, we suggest treating it as a primary path in the model; future research should focus on examining the mediating role of anxiety symptoms in the relationship between cognition and suicidal ideation.

### 4.4. Serial Mediation: Implicit Health Beliefs → Lifestyle Behaviors → Anxiety Symptoms → Suicidal Ideation

Bandura’s social cognitive theory of self-regulation emphasizes that human behavior is shaped by self-monitoring and evaluating one’s ability to regulate in response to distress by comparing oneself against personal standards and environmental conditions (Bandura, 1991). Within this framework, implicit health beliefs and self-regulation may operate at distinct yet complementary levels. Implicit health beliefs reflect broader assumptions about the controllability of health and may shape individuals’ assessments of health-related difficulties as well as their perceptions of the feasibility of behavioral change. The self-regulation process, in turn, may influence how individuals monitor their responses to these difficulties, evaluate feasible courses of action, and translate beliefs and goals into behavior. Consequently, weaker incremental health beliefs may predispose individuals toward less adaptive assessments and behavioral responses, whereas stronger incremental beliefs may promote engagement in health-promoting behaviors (Thomas et al., 2019).

Consistent with this theoretical perspective, physical activity, sedentary behavior, and sleep are associated with emotional well-being and anxiety symptoms (Philippot et al., 2022; Zhou et al., 2021), and anxiety symptoms are associated with suicidal ideation (Langer et al., 2024). Accordingly, maladaptive health beliefs may therefore be associated with less favorable behavioral patterns, which in turn may coincide with greater anxiety symptoms and suicidal ideation. However, this study did not directly measure these mechanisms; therefore, they should be regarded as theoretical explanations rather than empirically validated pathways. Furthermore, although the sequential indirect effect was statistically significant, its contribution was relatively small and should not be interpreted as the primary pathway in the model. Given the cross-sectional study design, the temporal sequence of the proposed sequence cannot be determined. Therefore, these findings merely provide a possible theoretical framework for explaining the observed sequential associations.

### 4.5. Strengths and Limitations

#### 4.5.1. Strengths

To our knowledge, this study is among the first to examine the indirect associations linking implicit health beliefs, lifestyle behaviors, anxiety symptoms, and suicidal ideation.

#### 4.5.2. Limitations

1. The cross-sectional study design limits causal inferences. Although the observed mediating relationship is consistent with the proposed theoretical model, it is not possible to establish the temporal sequence or causality. Therefore, the results of the mediation analysis should be interpreted as associations rather than causal pathways. Future longitudinal studies using repeated-measures designs are needed to further validate the proposed sequential mediation model.
2. Key variables were measured through self-reports, which may have introduced recall bias. Although efforts were made to control for potential confounding factors, residual or unmeasured confounding factors cannot be completely ruled out. Furthermore, convenience sampling within strata and voluntary participation may have introduced selection bias, as may differ systematically from non-participants. Additionally, all participants in this study were from a comprehensive medical university in Fujian Province, which limits the external validity and generalizability of the findings to college students in other regions and institutions across China.
3. Because the questionnaire included sensitive items assessing suicidal ideation, this study lacked a standardized post-survey psychological distress intervention protocol. Future studies should incorporate standardized suicide risk management procedures, including crisis resources and appropriate referral pathways, to better protect participants experiencing psychological distress.

## 5. Conclusions

This study provides preliminary empirical support for a theoretical model, the results of which suggest that lifestyle behaviors and anxiety symptoms may serve as potential mediating pathways in the relationship between implicit health beliefs and suicidal ideation. These findings highlight the importance of implicit health beliefs, lifestyle behaviors, and anxiety symptoms as potential areas of focus in suicide prevention and mental health promotion among college students. However, given that this study employed a cross-sectional design, these findings should not be interpreted as evidence of causality or temporal sequence; longitudinal and experimental studies are necessary to further test the proposed model.

## Figures and Tables

**Table 1:** Comparison of suicidal ideation scores by demographic characteristics.

Variables	Total Sample (n = 1964)	Suicidal Ideation				
		Mean (SD)	Test Statistics	Degrees of Freedom	Effect Sizes (d/$\eta^{2}$)	*p*-Value ^b^
Gender			−2.045	1962	0.090	<0.05
Male	777 (39.6)	0.88 (1.183)				
Female	1187 (60.4)	0.99 (1.272)				
Grade level			7.419	(3/1960)	0.011	<0.001
Freshman	711 (36.2)	0.81 (1.094)				
Sophomore	502 (25.6)	1.06 (1.241)				
Junior	404 (20.6)	1.12 (1.375)				
Senior	347 (17.7)	0.88 (1.313)				
Academic discipline			10.126	(2/1961)	0.010	<0.001
Medicine	1047 (53.3)	0.84 (1.128)				
Science/Technology	650 (33.1)	1.12 (1.336)				
Management/Law	267 (13.6)	0.96 (1.359)				
Residence			21.493	(3/1960)	0.032	<0.001
Natural village	319 (16.2)	1.36 (1.384)				
Township	549 (28.0)	1.06 (1.276)				
County township	478 (24.3)	0.75 (1.072)				
Main city of prefecture	618 (31.5)	0.79 (1.185)				
Monthly living expenses			30.323	(4/1959)	0.058	<0.001
≤1000 Yuan	134 (6.8)	1.93 (1.525)				
1001~1500 Yuan	615 (31.3)	1.06 (1.229)				
1501~2000 Yuan	678 (34.5)	0.85 (1.169)				
2001~2500 Yuan	348 (17.7)	0.71 (1.140)				
>2500 Yuan	189 (9.6)	0.67 (1.091)				
Average monthly household income ^a^			35.939	(3/1955)	0.052	<0.001
<2000 Yuan	262 (13.3)	1.65 (1.496)				
2000~4999 Yuan	565 (28.8)	0.95 (1.147)				
5000~9999 Yuan	695 (35.4)	0.78 (1.147)				
≥10,000 Yuan	437 (22.3)	0.79 (1.186)				

SD = standard deviation. ^a^ Missing of average monthly household income: 5. ^b^ One-way analysis of variance (ANOVA) was used to test differences in suicidal ideation scores across different demographic groups.

**Table 2:** Anxiety symptoms, implicit health beliefs, suicidal ideation, and lifestyle behaviors correlation analysis.

	AS	IHB	SI	LB
AS	1			
IHB	−0.340 **	1		
SI	0.471 **	−0.266 **	1	
LB	−0.160 **	0.198 **	−0.166 **	1

** *p* < 0.01. IHB = implicit health beliefs, SI = suicidal ideation, LB = lifestyle behaviors, AS = anxiety symptoms.

**Table 3:** Analysis of the serial mediation model of implicit health beliefs on suicidal ideation.

Variables	LB		AS		SI	
	β	*t*	β	*t*	β	*t*
IHB	0.0470	7.8106 ***	−0.6055	−14.6796 ***	−0.0562	−5.3811 ***
LB			−0.3524	−2.3066 *	−0.1035	−2.8136 **
AS					0.1063	19.5275 ***
R^2^	0.1100		0.1779		0.2695	
F	34.4541		52.7381		79.8918	

* *p* < 0.05, ** *p* < 0.01, *** *p* < 0.001. IHB = implicit health beliefs, SI = suicidal ideation, LB = lifestyle behaviors, AS = anxiety symptoms.

**Table 4:** A test of the serial mediation effect of implicit health beliefs on suicidal ideation.

Pathways	Coefficient	Std. Err.	95%CI	Percentage
IHB—LB—SI	−0.0049	0.0018	(−0.0086, −0.0015)	3.85%
IHB—AS—SI	−0.0644	0.0063	(−0.0770, −0.0523)	50.63%
IHB—LB—AS—SI	−0.0018	0.0008	(−0.0035, −0.0002)	1.42%
Total indirect effect	−0.0710	0.0065	(−0.0841, −0.0586)	55.82%
Direct effect	−0.0562	0.0104	(−0.0767, −0.0357)	44.18%
Total effect	−0.1272	0.0107	(−0.1482, −0.1062)	100.00%

IHB = implicit health beliefs, SI = suicidal ideation, LB = lifestyle behaviors, AS = anxiety symptoms.

## Data Availability

The datasets used and/or analyzed during the current study are available from the corresponding author on reasonable request. The data are not publicly available due to privacy concerns.

## Supplementary Materials

The following supporting information can be downloaded at: https://www.mdpi.com/article/10.3390/bs16091619/s1, Table S1: Actual enrollment, target percentage, final valid sample sizes and actual percentage.

## Abbreviations

24HMBQ	24 h Movement Behaviors Questionnaire
GAD-7	7-item Generalized Anxiety Disorder Scale
