# Supplementary material for: Implicit Health Beliefs and Suicidal Ideation in Chinese College Students: The Serial Mediating Roles of Lifestyle Behaviors and Anxiety Symptoms
Source: Behav Sci (Basel). 2026 Sep 10;16(9):1619. doi: 10.3390/bs16091619 (PMC13603336; doi:10.3390/bs16091619)
Supplement: Supplementary file 1 [file behavsci-16-01619-s001.zip › behavsci-4508190-supplementary.pdf]

Table S1 Actual enrollment, target percentage, final valid sample sizes and actual percentage

Grade level	Academic discipline	Actual enrollment numbers	Target percentage	Final valid sample sizes	Actual percentage
	Medicine	1570	15.04%	359	18.28%
Freshman	Science/Technology	890	8.53%	230	11.71%
	Management/Law	150	1.44%	122	6.21%
	Medicine	1474	14.12%	325	16.55%
Sophomore	Science/Technology	971	9.30%	141	7.18%
	Management/Law	130	1.25%	36	1.83%
	Medicine	1564	14.98%	158	8.04%
Junior	Science/Technology	890	8.53%	215	10.95%
	Management/Law	147	1.41%	31	1.58%
	Medicine	1581	15.15%	205	10.44%
Senior	Science/Technology	925	8.86%	64	3.26%
	Management/Law	146	1.40%	78	3.97%
	Total	10438	100.00%	1964	100.00%
